# Insecticide resistance in *Aedes aegypti* and *Aedes albopictus* in southern Benin: quantification, investigation of *kdr* mutations, and detection of detoxification enzyme activity

**DOI:** 10.1186/s41182-025-00875-6

**Published:** 2025-12-11

**Authors:** Alphonse Keller Konkon, Rock Aikpon, Isidore Hoyochi, David Mahouton Zoungbédji, Arthur Sovi, Albert Sourou Salako, Camus Konkon, Brice Dangnon, Geoffroy Yahoue, Romuld Victoir Adjovi, Lokossou Antoine, Juvenal Ahouandjinou, Nsele Kisambu Grace, Bruno Adjottin, Lamine Baba-Moussa, Razaki Osse, Martin Akogbéto, Germain Gil Padonou

**Affiliations:** 1 Centre de Recherche Entomologique de Cotonou (CREC), Cotonou, 06 BP 2604 Benin; 2 Faculté Des Sciences Et Techniques de L, Université d’Abomey-Calavi, Abomey-Calavi, Benin; 3 Laboratory of Biology and Molecular Typing in Microbiology, Department of Biochemistry and Cellular Biology, Abomey-Calavi, Benin; 4 Faculty of Agronomy, University of Parakou, Parakou, Benin; 5 Africa Youth Scientists Network, Cotonou, Bénin; 6 National Malaria Control Program, Cotonou, Bénin; 7 Institut Supérieur Pédagogique de Kikwit, Kikwit, Democratic Republic of the Congo

## Abstract

**Background:**

Insecticide resistance in arbovirus vectors threatens the effectiveness of vector control strategies in many endemic regions. Understanding resistance profiles and identifying underlying mechanisms are essential for preventing operational failures. This study assessed the susceptibility of *Aedes aegypti* and *Aedes albopictus* to commonly used insecticides in southern Benin and investigated the contribution of knockdown resistance mutations and metabolic detoxification pathways.

**Methods:**

Entomological surveillance was conducted in Lokossa, Bohicon, and Dassa, where ovitraps were used to collect eggs of *Aedes* species. Eggs were reared to the adult stage under controlled insectary conditions. Nonblood-fed females aged 2–5 days were tested using standard tube assays to determine susceptibility to deltamethrin at 0.05%, permethrin at 0.75%, and bendiocarb at 0.1%. Additional concentrations at fivefold and tenfold the diagnostic doses were used to measure resistance intensity. Polymerase chain reaction assays were performed to detect mutations in the voltage-gated sodium channel gene associated with knockdown resistance. Biochemical assays were conducted to quantify the activity of oxidases, glutathione-S-transferases, and esterases. Exact binomial tests were used to compute confidence intervals for mortality rates and allele frequencies.

**Results:**

*Aedes aegypti* populations from the three communities showed resistance to deltamethrin and permethrin at the diagnostic dose while remaining fully susceptible to bendiocarb. Mortality increased substantially at elevated concentrations, indicating moderate resistance intensity. *Aedes albopictus* populations were fully susceptible to all tested insecticides. Three mutations in the voltage-gated sodium channel gene, F1534C, V1016G, and S989P, were identified in *Aedes aegypti* with allele frequencies ranging between 33.89% and 46.67%. Biochemical assays revealed elevated oxidase activity in all *Aedes aegypti* populations, with increased levels of glutathione-S-transferases and both alpha- and beta-esterases in Bohicon and Lokossa.

**Conclusions:**

The study documents pyrethroid resistance in *Aedes aegypti* from southern Benin, while *Aedes albopictus* remains susceptible. Both species showed high susceptibility to bendiocarb. The presence of three knockdown resistance mutations at moderate frequencies together with increased detoxification enzyme activity indicates that both target-site and metabolic mechanisms contribute to resistance development. These findings underscore the need for integrated resistance management to preserve the effectiveness of insecticidal interventions.

## Introduction

Insecticide resistance among arbovirus vectors poses a major challenge to vector control efforts aimed at reducing disease incidence and ultimately achieving elimination. Recent studies have reported a significant increase in resistance levels, particularly to pyrethroids [1, 2], and have linked this trend to genetic mutations in vector populations [2, 3]. In West Africa, the public health situation has become increasingly concerning. Countries such as Burkina Faso have experienced recurrent dengue outbreaks in recent years, including reports of dengue-related deaths [4–6]. According to the World Health Organization's 2024 report, over half of the global dengue cases were reported in Burkina Faso alone [7]. Other West African nations, including Côte d’Ivoire, Mali, Senegal, and Nigeria, have also been substantially affected by the disease [8–13]. Owing to its geographical proximity to these countries, Benin is considered a high-risk area and warrants close attention from public health authorities In Benin, between July and August 2010, two dengue cases were diagnosed in travelers returning to Cotonou from France. In addition, in 2019, Benin reported 11 dengue cases in the municipalities of Cotonou, Porto-Novo, Sèmè-Kpodji, and Abomey-Calavi, including two fatalities among the confirmed cases (one in Abomey-Calavi and one in Porto-Novo) [14, 15]. Dengue cases were confirmed in the human population in Benin as early as 2019 [16]. Despite this, dengue continues to be classified as a neglected tropical disease, largely owing to insufficient surveillance and limited policy focus. In 2023, a nationwide study documented the widespread distribution and abundance of major arbovirus vectors in Benin [17]. Further investigation in 2024 revealed the circulation of three distinct dengue virus serotypes within local mosquito populations [18]. This cocirculation poses a heightened risk, as secondary infection with a different serotype has been associated with more severe manifestations of the disease, including dengue hemorrhagic fever and dengue shock syndrome [19, 20]. Given this context, strengthening entomological surveillance is critical for preventing or responding rapidly to potential outbreaks. This study was, therefore, designed to assess the current status of insecticide resistance in *Ae. aegypti* and *Ae. albopictus* populations in southern Benin and to evaluate the operational implications for vector control strategies. In addition to guiding local interventions, this work addresses a key knowledge gap regarding the resistance status of *Ae. albopictus* to insecticides in the West African region.

## Methodology

### Study sites

**Fig. 1:**
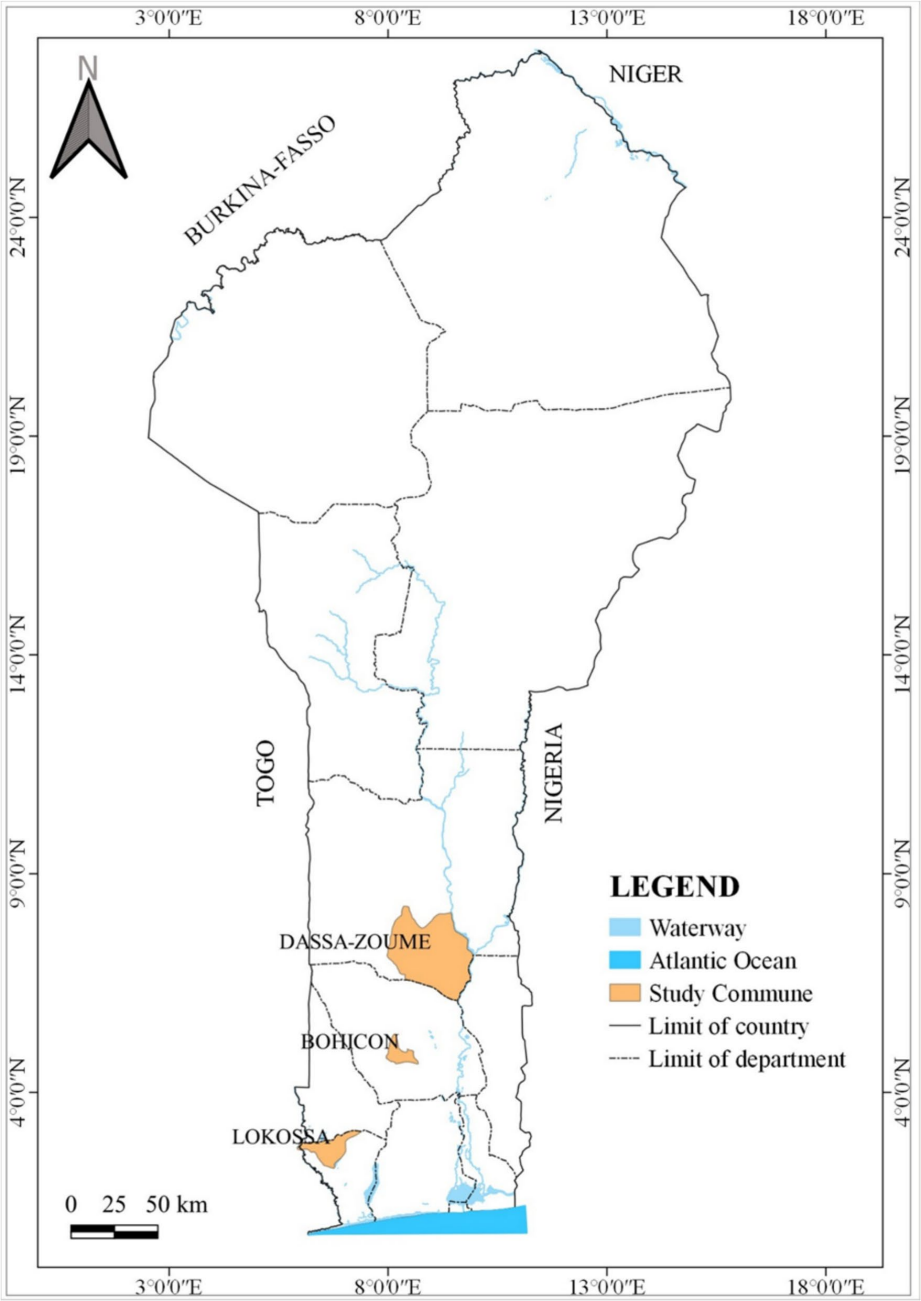
Study sites

The study was conducted in three communities of southern Benin: Lokossa (6°37′60″ N, 1°43′0″ E), Bohicon (7°11′00″ N, 2°04′00″ E), and Dassa-Zoumè (7°45′00″ N, 2°11′00″ E), as shown in the map of Benin (Fig. 1).

### Insecticide resistance status of *Ae. aegypti* and *Ae. albopictus* to pyrethroids and carbamates

#### Egg collection and mosquito rearing

Ovitraps were deployed in peridomestic environments across the three selected communities for a period of 7 days. The traps were then retrieved and transported to the insectary of the Centre de Recherche Entomologique de Cotonou (CREC). The collected eggs were immersed in water to stimulate hatching under favorable conditions. Following hatching, the larvae were reared under optimal insectary conditions (28 °C and ~ 80% relative humidity) [21]. Pupae were collected, pooled at the collection site, and transferred to holding cages for adult emergence. Adult mosquitoes were morphologically identified via taxonomic keys developed by Edwards and Huang [22, 23].

#### WHO tube bioassays for pyrethroids and carbamates

The susceptibility of female *Ae. aegypti* and *Ae. albopictus,* aged 2–5 days, to insecticides was assessed via the WHO tube test protocol. For each species, batches of 20 to 25 nonblood-fed females were aspirated from rearing cages and exposed for 60 min to diagnostic (1 ×), 5 × , and 10 × concentrations of deltamethrin, and permethrin, respectively. The diagnostic concentration of bendiocarb was used. The diagnostic concentrations (1 ×) of insecticide-impregnated papers used in WHO tube bioassays were as follows: 0.05% deltamethrin, 0.75% permethrin, and 0.1% bendiocarb. The corresponding increased concentrations used to assess resistance intensity were 5 × (0.25% deltamethrin; 3.75% permethrin) and 10 × (0.5% deltamethrin; 7.5% permethrin). During exposure, the number of mosquitoes whose expression was knocked down was recorded at 15-min intervals. The control groups (20–25 females per group) were simultaneously exposed to untreated filter papers to validate the test results. After exposure, the mosquitoes were transferred to observation tubes, provided with a sugar solution, and monitored for 24 h to determine mortality rates [24].

### Molecular and biochemical analyses

#### Detection of *kdr *mutations in *Ae. aegypti*

Genomic DNA was extracted from 30 individual *Ae. aegypti* samples per commune via the 2% CTAB protocol developed by the Centre de Recherche Entomologique de Cotonou (CREC). Sterile distilled water was added to the DNA pellet for resuspension. The reconstituted DNA was then used as a template for allele-specific PCR amplification to detect target-site mutations in the voltage-gated sodium channel (*vgsc*) gene. PCR was carried out using the following program: an initial denaturation and hot-start enzyme activation at 95 °C for 10 min, followed by 37 cycles of 94 °C for 30 s, 60 °C for 30 s, and 72 °C for 30 s, with a final extension at 72 °C for 7 min. [25]. Four kdr mutations were analyzed: S989P, V1016G, F1534C, and V1016I. The primers used for their detection are listed in Table 1.

**Table 1:** List of primers used for the detection of the *Kdr* mutation in *Ae. aegypti*

Mutation	Primers	Sequences (5′–3′)
S989P	M1-For	AATGATATTAACAAAATTGCGC
	M2-Rev	GCACGCCTCTAATATTGATGC
	M1-S	GCGGCGAGTGGATCGAAT
	M1-P	GCGGCGAGTGGATCGAAC
V1016G	M2-For	GCCACCGTAGTGATAGGAAATC
	M2-Rev	CGGGTTAAGTTTCGTTTAGTAGC
	M2-V	GTTTCCCACTCGCACAGGT
	M2-G	GTTTCCCACTCGCACAGGG
F1534C	M3-For	GGAGAACTACACGTGGGAGAAC
	M3-Rev	CGCCACTGAAATTGAGAATAGC
	M3-F	GCGTGAAGAACGACCCGA
	M3-C	GCGTGAAGAACGACCCGC
V1016I	Iso1016f	GCG GGC ACA AAT TGT TTC CCA CCC GCA CTG A
	Val1016f	GCG GGC AGG GCG GGG GCG GGG CCA CAA ATT GTT TCC CAC CCG CAC CGG

#### Biochemical assays

At each study site, thirty female *Ae. aegypti* mosquitoes, aged 2–5 days and not exposed to any insecticide, were selected for biochemical assays. The objective of this study was to quantify and compare the activity levels of major detoxification enzymes, including mixed-function oxidases (MFOs), non-specific esterases, and glutathione-S-transferases (GSTs), across field-collected populations relative to the insecticide-susceptible Rockefeller strain. The procedures followed the protocol established by Hemingway et al. [26]. Depending on the enzymatic activity measured, the following procedures were performed:

##### Glutathione-S-transferase (GST) activity

For the assay of GST activity, 10 µL of mosquito homogenate (in duplicate) was dispensed into each well of a Nunc microplate. To each well, 200 µL of a reaction mixture containing reduced glutathione (GSH) and 1-chloro-2,4-dinitrobenzene (CDNB) was added. After incubation at 25 °C, absorbance was recorded kinetically at 340 nm for 5 min using a spectrophotometer.

##### Oxidase activity

For oxidase determination, 20 µL of mosquito homogenate (in duplicate) was added to each well, followed by 80 µL of 0.0625 M potassium phosphate buffer (pH 7.2), 200 µL of 0.25 M tetramethylbenzidine (TMBZ, pH 5.0), and 25 µL of 3% hydrogen peroxide (H₂O₂) solution. The plate was incubated for 1 h, and absorbance was measured at 630 nm using a spectrophotometer.

##### Non-specific esterase activity

For non-specific esterases, 10 µL of mosquito homogenate (in duplicate) was placed into each well, followed by 90 µL of 1% Triton phosphate-buffered saline (PBS, pH 6.5). After mixing, the plate was left for 10 min at 25 °C (room temperature). Then, 100 µL of a substrate solution consisting of 0.3 M α-naphthyl acetate (or β-naphthyl acetate), 2.5 mL of Triton PBS (pH 6.5), and 7 mL of distilled water was added. The plate was incubated again for 30 min, after which 100 µL of Fast Garnet Salt (FGBC) solution was added. The mixture was incubated for an additional 10 min at 25 °C (covered), and absorbance was read at 550 nm using a spectrophotometer.

### Data analysis

Mortality was recorded 24 h after insecticide exposure, and the results were interpreted according to World Health Organization (WHO) guidelines [27].

- A mortality rate between 98% and 100% indicates that the tested mosquito population is fully susceptible to the insecticide.
- A mortality rate less than 98% suggests suspected resistance, which requires further investigation.
- A mortality rate less than 90% confirms the presence of resistance genes within the tested population.

For tests conducted with increased diagnostic concentrations (5 × and 10 ×), the following interpretations were applied:

- A mortality rate > 98% at a 5 × concentration indicates low resistance**.**
- A mortality rate > 98% at a 10 × concentration indicates moderate resistance.
- A mortality rate < 98% at a 10 × concentration indicates high resistance**.**

#### Allelic frequency estimation of *kdr* mutations

The frequency of *kdr* alleles was calculated via the basic principles of Mendelian genetics, which is based on the following formula:

$$F = \left( {2n{\text{RR}} + n{\text{RS}}} \right)/2\left( {n{\text{RR}} + n{\text{RS}} + n{\text{SS}}} \right)$$

where *n* represents the number of mosquitoes with each specific genotype (RR: homozygous resistant, RS: heterozygous, SS: homozygous susceptible).

This formula has been widely applied and popularized in entomological research by Martinez-Torres et al. [28].

Exact binomial tests were used to compute 95% confidence intervals for both mortality rates and *kdr* allelic frequencies.

To evaluate differences in enzyme activity across mosquito populations, a linear regression model coupled with ANOVA was applied. Comparisons between enzyme activity levels in field-collected populations and the reference Rockefeller strain were performed via the nonparametric Mann–Whitney *U* test.

## Results

### Diversity of culicids collected using ovitraps

The results obtained from ovitrap collections revealed a clear predominance of the genus *Aedes* across the three districts surveyed. *Aedes aegypti* was the most abundant species in Lokossa (58.55%), Bohicon (57.50%), and Dassa (62.33%), followed by *Aedes albopictus*, whose proportions ranged from 34.23% in Dassa to 41.36% in Bohicon. Species of the genus *Culex* were less represented, with *Culex quinquefasciatus* accounting for 1.14–3.44% and *Culex nebulosus* observed only in Lokossa (0.70%) (Table 2). The dominance of *Aedes*, as evidenced by eggs collected in ovitraps, indicates a high level of arbovirus vector activity in these urban and peri-urban areas and underscores the need to strengthen entomological surveillance and targeted vector control interventions.

**Table 2:** Species diversity of culicids collected using ovitraps

Districts			
Mosquitoes species	Lokossa % (n)	Bohicon % (n)	Dassa % (n)
*Aedes aegypti*	58,55 (3600)	57,50 (4383)	62,33 (2027)
*Aedes albopictus*	38,33 (2357)	41,36 (3153)	34,23(1113)
*Culex quinquefasciatus*	2,42 (149)	1,14 (87)	3,44 (112)
*Culex nebulosus*	0,70 (43)	0,00 (0)	0,00 (0)
Total	6149	7623	3252

### Susceptibility of *Ae. aegypti* and *Ae. albopictus* to pyrethroids and carbamates

**Fig. 2:**
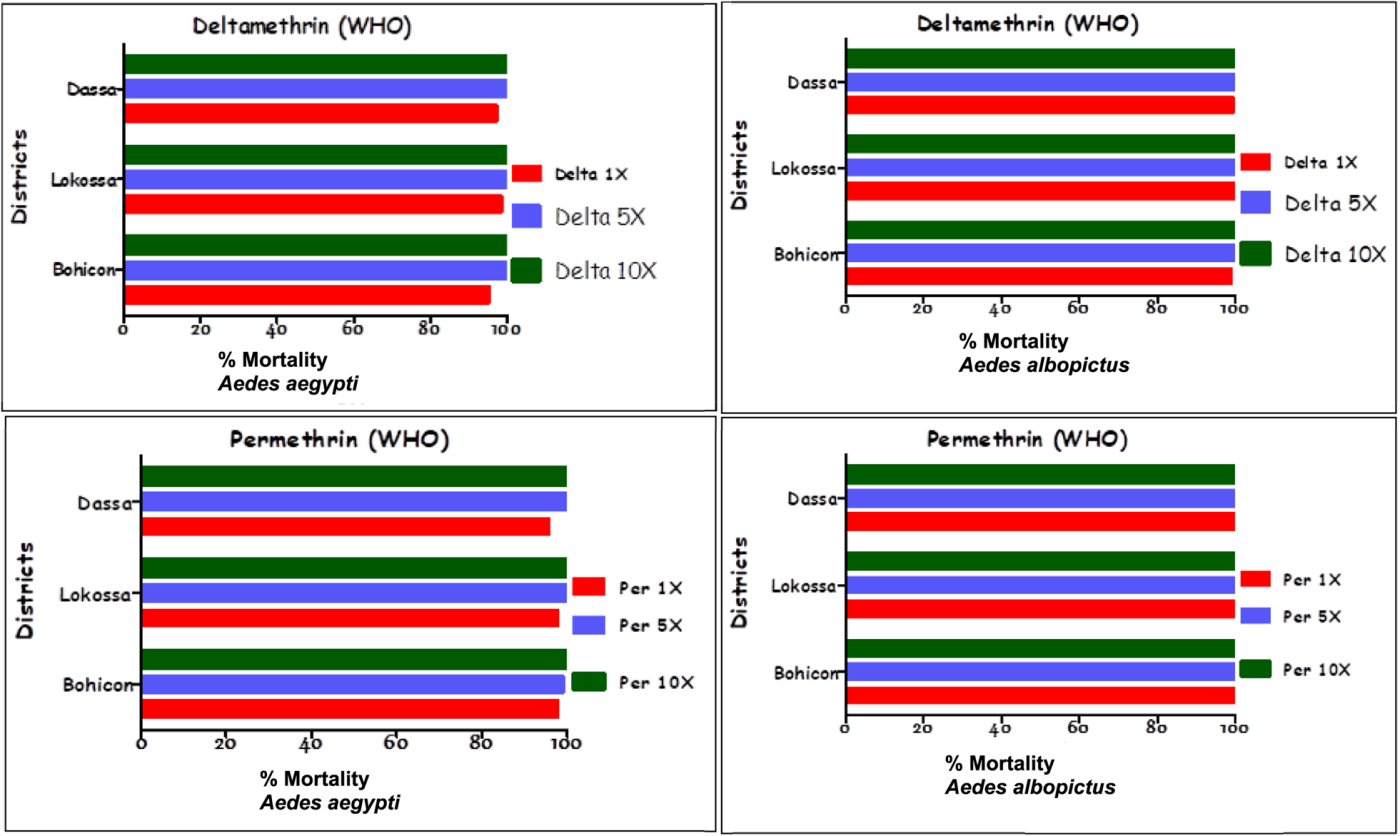
Mortality rates of *Ae. aegypti* and *Ae. albopictus* exposed to permethrin and deltamethrin

**Fig. 3:**
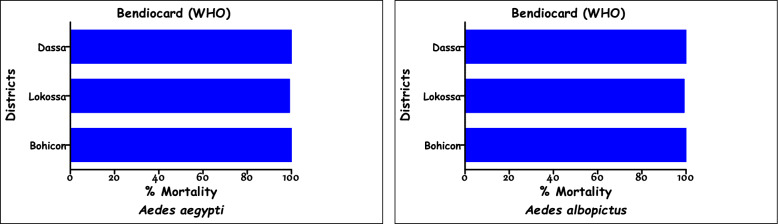
Mortality rates of *Ae. aegypti* and *Ae. albopictus* exposed to bendiocarb

Populations of *Ae. aegypti* and *Ae. albopictus* from Dassa, Lokossa, and Bohicon were exposed to two pyrethroids (deltamethrin and permethrin) and one carbamate (bendiocarb) at three concentrations (1 × , 5 × , and 10 ×) to assess their level of resistance to these insecticides. For *Ae. aegypti*, all populations exhibited resistance (mortality < 90%) when exposed to the diagnostic concentration (1 ×) of deltamethrin and permethrin (Fig. 2). However, high levels of susceptibility to bendiocarb at the same concentration were observed in Dassa (98.2%), Lokossa (99%), and Bohicon (100%) (Fig. 3). When exposed to relatively high concentrations (5 × and 10 ×), mortality rates increased, ranging from 99% to 100% with permethrin, and reached 100% with both deltamethrin and bendiocarb in all the tested populations. In contrast, *Ae. albopictus* populations from all three locations were fully susceptible (mortality > 98%) to the diagnostic concentrations (1 ×) of all three insecticides. Specifically, the mortality rates for deltamethrin were 99.5% in Dassa, 99.7% in Lokossa, and 98.8% in Bohicon. With permethrin, mortality reached 100% in both Dassa and Lokossa and 99.8% in Bohicon (Fig. 2). Bendiocarb induced mortality rates of 98.8%, 99.9%, and 100% in Dassa, Lokossa, and Bohicon, respectively (Fig. 3). Exposure to increased concentrations (5 × and 10 ×) of deltamethrin, permethrin, and bendiocarb resulted in 100% mortality across all *Ae. albopictus* populations (Figs. 2 and 3).

### Frequency of *kdr *mutations in *Ae. aegypti*

Three *kdr* mutations were detected across all study sites, with allelic frequencies varying between localities (Table 3). For the F1534C mutation, the frequency ranged from 23.33% [8.20–38.47] in Dassa to 43.33% in Bohicon, with an overall average of 33.89% [24.11–43.67] across all locations. These results may reflect a transitional phase in which local *Ae. aegypti* populations shift from susceptible to resistant. With respect to the V1016G mutation, allelic frequencies varied from 28.33% [12.21–44.46] in Dassa to 56.67% [38.93–74.40] in Lokossa. For the S989P mutation, the frequency ranged from 25.00% [9.50–40.50] in Dassa to 41.67% [24.02–59.31] in Bohicon, with an overall mean of 33.89% [24.11–43.67]. Overall, the mean allelic frequencies across all sites were 33.89% [24.11–43.67] for F1534C, 46.67% [36.36–56.97] for V1016G, and 33.89% [24.11–43.67] for S989P. No individuals carrying the V1016I mutation were detected at any of the study sites.

**Table 3:** Allelic frequencies of *kdr* mutations (S989P, V1016G, and F1534C) in *Ae. aegypti* populations from Dassa, Bohicon, and Lokossa

	N tested	RR	RS	SS	Kdr frequency	95% CI	
F15434C							
Dassa	30	1	12	17	23,33	8,20	38,47
Lokossa	30	1	19	10	35,00	17,93	52,07
Bohicon	30	2	22	6	43,33	25,60	61,07
All area	90	4	53	33	33,89	24,11	43,67
V1016G							
Dassa	30	1	15	14	28,33	12,21	44,46
Lokossa	30	4	26	0	56,67	38,93	74,40
Bohicon	30	7	19	4	55,00	37,20	72,80
All area	90	12	60	18	46,67	36,36	56,97
S989P							
Dassa	30	5	5	20	25,00	9,50	40,50
Lokossa	30	6	13	11	41,67	24,02	59,31
Bohicon	30	4	13	13	35,00	17,93	52,07
All area	90	15	31	44	33,89	24,11	43,67

### Assessment of oxidase, esterase, and GST activity in *Ae aegypti*

**Fig. 4:**
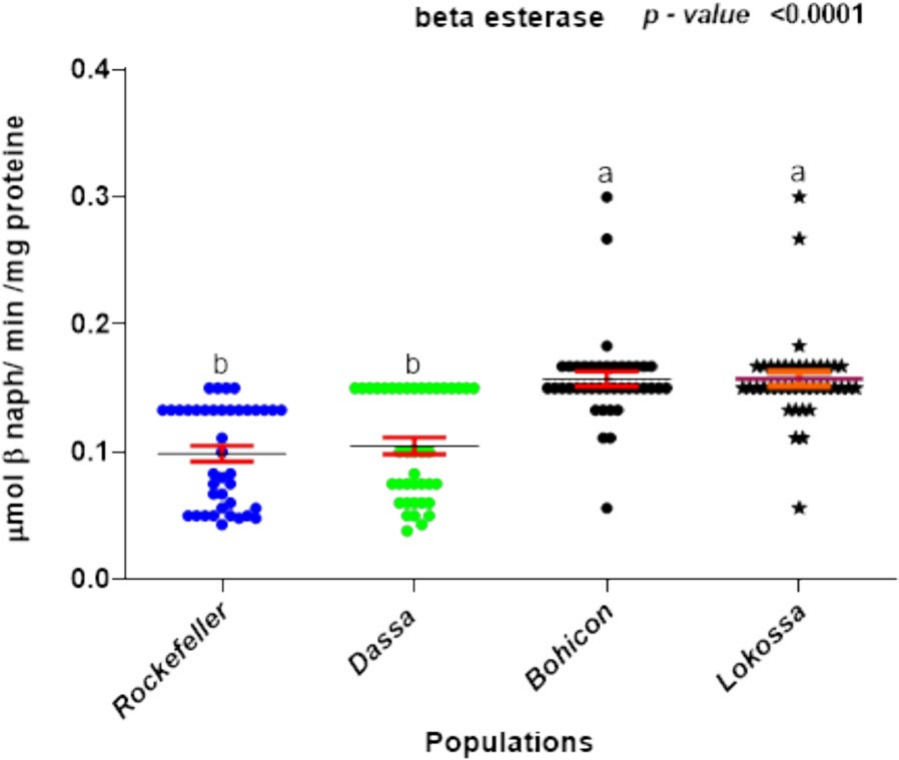
**a** Measurement of the activity of detoxification enzymes (oxidases) in *Ae. aegypti*. **b** Measurement of the activity of detoxification enzymes (GSTs) in *Ae. aegypti.* **c** Measurement of the activity of detoxification enzymes (alpha esterases) in *Ae. Aegypti*. **d** Measurement of the activity of detoxification enzymes (beta-esterases) in *Ae. aegypt*

Populations of *Ae. aegypti* collected from Dassa, Bohicon, and Lokossa presented significantly greater mixed-function oxidase activity than did the insecticide-susceptible Rockefeller reference strain. Glutathione-S-transferase (GST) activity was detected in all field populations, with particularly elevated levels observed in Bohicon and Lokossa. In addition, increased esterase activity was recorded in the Bohicon and Lokossa populations, where both α- and β-esterases showed significantly greater activity than in the Rockefeller strain. In contrast, no statistically significant difference in α- or β-esterase expression was detected between the Dassa population and the susceptible reference strain (Fig. 4a–d).

## Discussion

This study provides an overview of insecticide resistance in *Aedes* mosquitoes, the primary arbovirus vectors in Benin. It quantifies resistance levels to commonly used insecticides and explores the possible involvement of mutations in voltage-gated sodium channel genes. The results revealed that *Ae. aegypti* populations were resistant to the diagnostic doses (1 ×) of deltamethrin and permethrin, whereas *Ae. albopictus* remained fully susceptible. Both species were completely susceptible to the diagnostic dose of bendiocarb across all the sites. Furthermore, *Ae. aegypti* remained susceptible to increased doses (5 × and 10 ×) of deltamethrin and permethrin at all locations.

In the context of indoor residual spraying (IRS), high levels of resistance can compromise vector control efficacy, as the insecticide concentrations achieved on sprayed surfaces under field conditions are unlikely to reach those used in laboratory bioassays. Consequently, the persistence and bioavailability of pyrethroids on treated walls may be insufficient to induce lethal effects in resistant mosquito populations, potentially reducing the overall impact of IRS interventions. Furthermore, these findings underscore the need for regular monitoring of resistance intensity and the adoption of alternative or combination insecticides with different modes of action to sustain the long-term effectiveness of IRS programs [29]. Previous studies have linked such patterns to the presence of mutations in the voltage-gated sodium channel gene (commonly referred to as *kdr*) or to increased enzymatic detoxification mechanisms in mosquitoes [30–34]. The resistance of *Ae. aegypti* to pyrethroids has already been documented in Benin and other West African countries [1, 2, 25], where this species has developed increasing tolerance to pyrethroids, the most widely used class of insecticides in public health. In contrast, *Ae. albopictus*, more recently reported in Benin, still shows full susceptibility to pyrethroids [1, 35]. Both vectors were fully susceptible to bendiocarb, a carbamate insecticide. This difference could be explained by the distinct modes of action of the two insecticide classes: organophosphates and carbamates inhibit acetylcholinesterase, whereas pyrethroids act by disrupting the function of voltage-gated sodium channels. [36, 37]. In addition, the selective pressure for resistance to carbamates remains low, given their limited use in public health interventions (e.g., indoor residual spraying, ITNs) and relatively modest application in agriculture, where they represent only 16.6% of insecticide use in Benin [31, 38–40]. This study also reported the presence of three *kdr* mutations, F1534C, V1016G, and S989P, in *Ae. aegypti* populations. These mutations were detected at varying frequencies across sites, ranging overall from 33.89% to 46.67%. This heterogeneity suggests ongoing genetic shifts within *Ae. aegypti* populations, supporting the phenotypic resistance profiles observed in this study. The allelic frequencies are consistent with an early phase of resistance emergence or a transitional stage from susceptibility to resistance [2, 32]. The partial restoration of susceptibility to permethrin and deltamethrin at relatively high concentrations might be due to the relatively low *kdr* allele frequencies, indicating moderate resistance levels [31, 41]. These findings highlight the importance of closely monitoring the frequencies of the *F1534C*, *V1016G*, and *S989P* alleles to track the potential evolution of resistance before allele fixation occurs. The high levels of mixed-function oxidase (MFO) activity detected in *Ae. aegypti* from Dassa, Bohicon, and Lokossa further support the involvement of oxidative metabolic pathways in pyrethroid resistance, a well-documented mechanism linked to vector control failure [42, 43]. Elevated GST activity, particularly in Bohicon and Lokossa, can contribute to pyrethroid detoxification, a well-established mechanism. This observation aligns with previous reports from West Africa suggesting a growing role of GSTs in resistance to conventional insecticides [44]. Moreover, the elevated activity of α- and β-esterases observed in *Ae. aegypti* populations from Bohicon and Lokossa indicates enhanced metabolic activity, although it does not appear sufficient to confer phenotypic resistance to bendiocarb, to which these populations remained fully susceptible. This suggests that these esterases may play a broader detoxification role, potentially involved in cross-resistance to organophosphates rather than directly mediating carbamate resistance [39, 45]. Although increased esterase activity was observed in certain *Aedes aegypti* populations, this biochemical response does not necessarily confer phenotypic resistance to carbamates, such as bendiocarb. The observed susceptibility may indicate that the level or isoform of esterases expressed is inadequate to catalyze the hydrolysis of carbamate molecules efficiently, or that these enzymes are preferentially involved in the detoxification of other insecticide classes, particularly organophosphates. Alternatively, the upregulation of esterase genes might reflect a generalized metabolic adjustment, serving a supportive rather than a direct role in resistance mechanisms. These results underscore that elevated esterase activity should not be regarded as definitive evidence of carbamate resistance, but rather as part of the broader biochemical plasticity that enables mosquito populations to cope with diverse insecticidal pressures. The detection of pyrethroid resistance in *Aedes aegypti*, despite the moderate occurrence of *kdr* mutations and the variable expression of detoxification enzymes among sites, indicates that several mechanisms may interact either synergistically or independently to produce the observed resistance phenotype. In certain areas, such as Dassa, the metabolic profile suggests that oxidases or esterases play a more prominent role, whereas in other locations, resistance appears to be primarily driven by *kdr*-associated target-site insensitivity. This spatial variability underscores the complex and multifactorial nature of pyrethroid resistance, where both metabolic detoxification and target-site modifications may act in a compensatory manner, influenced by local selection pressures, mosquito genetic background, and the intensity of insecticide exposure. These findings underscore the urgent need for continued resistance surveillance to anticipate potential failures in vector control programs. Given the confirmed pyrethroid resistance in *Ae. aegypti*, alternative insecticides like bendiocarb could be considered for emergency space spraying during outbreaks, while long-term strategies should invest in non-chemical methods. This study has several limitations. First, it was conducted in only three municipalities, limiting the generalizability of its findings at the national level. Second, it was not a longitudinal study and thus did not capture resistance dynamics over time. Nevertheless, it provides a critical snapshot of the current resistance status and emphasizes the need for ongoing monitoring to prevent the spread of resistance across *Aedes* populations in Benin.

## Conclusion

This study confirms the resistance of *Ae. aegypti* to the diagnostic doses of permethrin and deltamethrin in the municipalities of Lokossa, Bohicon, and Dassa-Zounmè, while *Ae. albopictus* remains fully susceptible. Both species were susceptible to bendiocarb. The detection of *kdr* mutations F1534C, V1016G, and S989P in all sites, at frequencies indicative of emerging resistance, highlights a concerning trend. In addition, detoxification enzymes such as oxidases, GSTs, and esterases (α and β) were identified in *Ae. aegypti*, underscoring the metabolic component of insecticide resistance in these populations. These findings provide an essential baseline for establishing continuous resistance monitoring frameworks and for guiding adaptive vector control strategies aimed at preventing the future spread of insecticide resistance in *Ae. albopictus* populations.

## Data Availability

The data sets analyzed in this study are available from the corresponding authors upon reasonable request.

## Abbreviations

CREC: Centre de Recherche Entomologique de Cotonou
WHO: World Health Organization
MFO: Mixed-function oxygenases
GST: Glutathione-S-transférase
IRS: Indoor residual spraying
